# 

**Published:** 1851-11

**Authors:** 


### ARTICLE IV.

### NOTICE TO READERS AND CORRESPONDENTS.

We have received original communications from Drs. W. Matthews and J. E. McGirr.

We have received from Messrs. Blanchard & Lea of Philadelphia, through S. C. Griggs & Co., Booksellers of Chicago, the following books for notice, which shall receive attention as early as possible: A new edition of Churchill's Midwifery; Gross on the Diseases and Injuries of the Urinary Bladder, Prostrate Gland, and the Urethra; Letters to a can did Inquirer on Animal Magnetism by Prof. W. Gregory; Beale on the Laws of Health; Walshe on the Heart and the Lungs and Bird on Urinary Deposit. We have also received the Southern Medical Reports (in exchange). The Transactions of the Medical Society of the State of Pennsylvania. The Proceedings of the Illinois State Medical Society. A Circular pamphlet to the Medical Profession by Dr. H. A. Ramsay of Raysville, Ga. The New Orleans Monthly Medical Register, No. 1., Vol. 1., for October. An extra of the Western Journal of Medicine and Surgery, being a crontroversial reply to Prot. Bullitt. The proceedings of the Iowa State Medical and Chirurgical Society, at its second annual meeting in May last. A small paper called the Belmont Farmer, asking how we will exchange, to which we reply for $2 per annum in advance. We have also received our usual list of exchanges.

### RECEIPTS FOR THE JOURNAL FOR SEPTEMBER AND OCTOBER.

ILLINOIS.

| | |
|---|---|
| Dr. W. H. Williams, | $4 00 |
| Richd' McGee, | 2 00 |
| O. S. Johnson, | 2 00 |
| J. H Lyford, | 2 00 |
| A. W. Rawson, | 4 00 |
| S. M. Reynolds, | 2 00 |
| J. B. Hunt, | 2 00 |
| P. Reinbold, | 2 00 |
| WISCONSIN. | |
| Dr. J. M. Todd, | 2 00 |
| Francis Paddock, | 2 00 |
| MICHIGAN. | |
| Dr. J. S. Skinner, | 2 00 |
| N. M. Thomas, | $2 00 |
| H. Knapp, | 2 00 |
| B. P. Wells, | 2 00 |
| INDIANA. | |
| Dr. J. Q. A. Banta, | 4 00 |
| J. G. Orsborne, | 3 00 |
| D. W. Skinner, | 2 00 |
| Clay Brown, | 2 00 |
| W. H. Martin, | 6 00 |
| Solomon Simpson, | 4 00 |
| E. Van Sossen, | 2 00 |
| OHIO. | |
| Dr. P. H. Clarke, | 5 00 |

### TO SUBSCRIBERS.

We are not fond of complaining, but duty sometimes compels us to speak of unpleasant things, in order that they may be bettered. We are now, in consequence of the miserably short list of receipts above, obliged to put those who take the Journal without paying promptly for it, in mind that we want our pay. We think it unfair that they keep us out of our money when we have to pay the cash for the printing and paper of every number, before we can send it to them.

Please accommodate us, by remitting at once.

